# Nutrients and rheumatoid arthritis: From the perspective of neutrophils

**DOI:** 10.3389/fimmu.2023.1113607

**Published:** 2023-02-27

**Authors:** Ya-Ru Shao, Dan-Yi Xu, Jin Lin

**Affiliations:** Department of Rheumatology, The First Affiliated Hospital, Zhejiang University School of Medicine, Hangzhou, China

**Keywords:** nutrients, rheumatoid arthritis, neutrophil, neutrophil extra cellular traps, inflammation

## Abstract

Neutrophils are considered as core immune cells involve in the early stage of rheumatoid arthritis (RA) and participate in the disease progression. The underlining mechanisms include the elevated chemotaxis and infiltration of neutrophils, the increase in the reactive oxygen species and the promotion of neutrophil extracellular traps formation. Accumulating studies demonstrated the important role of nutrients intake played in the initiation and progression of RA. This study summarized the effects of several macronutrients and micronutrients on regulating RA through the modulation of activated neutrophils and appealed for a healthy diet in RA-risk individuals as well as RA patients.

## Introduction

Rheumatoid arthritis (RA) is one of the most common systemic autoimmune disease, with a worldwide prevalence of around 0.5% (1). This is a chronic progressive disease mostly characterized by synovitis. Autoantibodies and immune cells infiltrate and cumulate in the synovial cavity, causing arthralgia, bone destruction and finally joint deformity. The etiology of RA is complex and not thoroughly clarified, with both susceptibility genes and environmental risk factors involved. Over the past decades, in spite of the decreased mortality, RA has been more and more prevalent especially in developed countries and urban areas, accompanied by earlier disease onset, which emphasizes the importance of environmental triggers (2). Mucosal sites, such as respiratory tracts and intestinal tracts, are thought to be the places where inflammation initially occurs in the hypothesis of ‘mucosal origin’ (3). This hints that air and food may be the most relevant source of environmental triggers. In fact, smoking has been deemed as the most important environmental factor in the risk of RA (4).

On the other side, immune imbalance is regarded as a critical part in the pathogenesis of RA and interacts with environmental factors. The excessive recruitment of neutrophils is thought to be crucial for the initiation of RA and also participate in the progression and perpetuation of RA through several mechanisms. There exists a rising interest on whether nutrients consumption participates in the pathogenesis and progression of RA through modulating the activity of neutrophils. In this study, we aimed to explore and summarize the function of nutrients in regulating RA, especially focusing on how they modulate the infiltration and activation of neutrophils.

## Neutrophils in the pathogenesis of RA

In the process of inflammation, neutrophil is the first kind of immune cell recruited *via* chemotaxis, and always remains to take the highest proportion in the inflamed sites (5). Under normal conditions, neutrophils have a relative short half-life less than 1 day. Whereas during inflammation, activated neutrophils acquire severalfold prolonged life spans (6, 7). As one of the most important cell types in the immune system, neutrophil can defend against pathogens directly by phagocytosis or by releasing granular enzymes such as myeloperoxidase (MPO), matrix metalloproteinase (MMP) and neutrophil elastase (NE). It can also produce reactive oxygen species (ROS) *via* the activation of membrane-bound NADPH-oxidase, thus promote respiratory burst. Furthermore, the networks that neutrophil forms with granular enzymes and extracellular nuclear contents, nominated as neutrophil extracellular traps (NETs), are able to entrap and eliminate pathogens with great efficiency (8, 9).

Supported by the results of proteomic analysis, neutrophil is the most abundant cell type in the inflamed synovial fluid of RA (10). Neutrophils in the RA synovial fluid presented with elevated expression of chemokines, which further amplified the inflammatory response. In addition, those neutrophils also produced more ROS, exacerbating oxygen stress (11). There was also a strengthened activation of NETs, together with postponed apoptosis (12). Furthermore, anti-citrullinated protein antibodies (ACPAs), which are markable in RA, are considered to be associated with activated neutrophils through the exposure of related antigens in the NETs (13, 14).

## Nutrients exacerbate inflammation

### Carbohydrates

In a case-control study which retrospectively assessed the diet consumed 5 years before disease onset based on the Chinese population, RA patients consumed more carbohydrates than healthy controls. Increased carbohydrates intake might make excessive energy absorption, lead to increased body weight and elevate the risk of RA (15, 16).

More pieces of evidence were in the case of monosaccharide. An observational study indicated that the intake of sugar-sweetened soda increased the risk of RA (17). Researchers also found an association between the consumption of high-fructose soft drinks and the onset of RA (18). The background mechanism may be that elevated glucose and fructose ingestion promote the production of advanced glycation end products (AGEs) and enhance autophagy and NETosis (19). Whereas, a recent cohort study based on the French population found no correlation between sugar-sweetened soft drinks and RA risk, but indicated that artificially-sweetened soft drinks increased RA risk (20).

### Glutamine

Glutamine, which is a non-essential amino acid relatively abundant in beef, eggs, tofu and other protein-rich foods, is another source of energy in addition to glucose. Glutamine is consumed at the highest rate by neutrophils compared with other immune cells (21). As the substrate of NADPH, glutamine participants in increasing superoxide generation through NADPH oxidase in neutrophils (22). Besides, glutamine is involved in the synthesis of O-linked beta-N-acetylglucosamine (O-GlcNAc), which is increased in activated neutrophils and promotes cellular mobility *via* the MAPK pathway (23). However, there were rare publications focused on the association of glutamine supplementation and RA, except for one study conducted 17 years ago which showed that supplementation with beta-hydroxy-beta-methylbutyrate, glutamine and arginine had no benefit in reversing cachexia in RA patients (24).

### Red meat

Red meat may exacerbate inflammation through saturated fatty acids and nitrites. But whether red meat intake can increase the risk of RA is still under debate. In a case-control study, a high red meat intake was associated with an increase in the risk of inflammatory polyarthritis (25). Compared to a meat-rich diet, people consuming a vegan diet experienced a decrease in total neutrophil counts (26). In RA patients who underwent a 3-month diet excluding meat, gluten and lactose, the circulating neutrophils were significantly decreased, together with a relief of inflammation symptoms (27). And in a recent cross-sectional study with 707 RA patients recruited, a high intake of red meat was associated with earlier disease onset, especially in those with smoking habits or overweight problems (28). Nevertheless, there were also lots of studies suggesting no effect red meat consumption laid on the risk of RA (29, 30). A recent meta-analysis analyzed 7 cohorts and 6 case-control studies also found no significant association between red meat consumption and the risk of RA (31).

## Nutrients ameliorate inflammation

### Omega-3 fatty acid

Omega-3 polyunsaturated fatty acid was considered as a protective factor against RA. Eicosapentaenoic acid (EPA) and docosahexaenoic acid (DHA), which are rich in deep-sea fishes, have been shown to be able to inhibit NF-κB signal, thus reducing the production of pro-inflammatory cytokines (32). With the suppression of chemotaxis, the recruitment and infiltration of leucocytes were also inhibited (33). The metabolic product of omega-3, resolvin, was also found able to attenuate inflammation and relieve joint pain *via* the inhibition of neutrophil recruitment in RA (34). A prospective cohort study showed that a more than 0.21 g per day dietary consumption of long-chain omega-3 polyunsaturated fatty acids was associated with a 35% decline in the risk of RA (35). In a cross-sectional study, fish consumption no less than 2 times per week was able to attenuate the disease severity of RA patients (36).

### Vitamin D

Previous reports have found that RA patients experience significantly lowered levels of 25-hydroxyvitamin D [25(OH)D] (37, 38). And the deficiency of 25(OH)D was thought to be associated with a higher disease severity (39). The seasonal fluctuation of disease performance might be related to the seasonal variation of the serum 25(OH)D levels (40).

In fact, vitamin D (vit D) took part in the amelioration of inflammation by reducing the synthesis of pro-inflammatory mediators, inhibiting the release of ROS, and decreasing NETosis (41, 42). In mouse models, supplementation of Vitamin D3 was able to promote ATP degradation and revert E-ADA activity in neutrophils, thus ameliorating the joint symptoms (43). And in RA patients with vit D deficiency, supplementation of vit D could rapidly improve the disease activity (44). Moreover, five years of vit D supplementation could reduce the incidence of autoimmune disease by 22% (45).

### Zinc

The serum zinc level is also decreased in RA, probably because of the increased zinc import to the cell under the exposure of pro-inflammatory cytokines (46). As a result, the deficiency of zinc will further promote inflammation by increasing the release of pro-inflammatory mediators and ROS by epigenetic mechanisms (47).

In mice models, supplementation of zinc by injection could decrease the recruitment and activity of neutrophils, thus ameliorating inflammation and tissue damage (48). A meta-analysis of clinical trials indicated that with the increase of serum zinc, neutrophil levels decreased, and so was circulating CRP, hs-CRP, TNF-α and IL-6 (49). However, as for the effect zinc supplementation laid on NETosis, there remains a controversy. Some reports suggested that zinc could inhibit NETosis by inhibiting histone citrullination (48, 50). While some others found an increase in the NETs formation and release after the treatment of zinc (51, 52).

### Selenium

Selenium is another trace element in the human body and showed remarkable anti-inflammation and antioxidant potential in RA. Compared to normal controls, patients with RA presented with significantly lower serum selenium levels (53). Moreover, RA patients with higher serum selenium concentration seemed to have milder inflammation, indicated by lower levels of CRP and ESR (54). Animal studies proposed that selenium-treated RA mice presented with reduced neutrophil counts, decreased NETs production, downregulated pro-inflammatory cytokines, and improved disease severity (55). Additionally, selenium also caused a reduction of ROS and alleviated oxidative stress (56).

### Ferrum

Iron plays an important role both in the recruitment and in the physical functions of neutrophils. Compared to normal controls, the serum iron level was significantly lower and the level of soluble transferrin receptor was elevated in RA patients (57). RA aggravated iron redistribution, making a decrease of iron in the blood and an increase in the synovium, and amplifying local inflammation (58). Iron imbalance contributed to RA inflammation. On the one hand, a deficiency of iron might promote the formation of NETs, which could be reversed by iron supplementation. On the other hand, when it came to iron overload, free iron would participate in NETosis and increase inflammation, which was able to be rescued by iron chelators (59).

### N-acetylcysteine

N-acetylcysteine (NAC) is the acetylated form of L-cysteine. The supplementation of NAC is widely utilized in chronic obstructive pulmonary disease and acetaminophen intoxication, but has not been recommended in RA (60). NAC could remove ROS and inhibit the synthesis of pro-inflammation cytokines, thus reducing the recruitment of neutrophils and other immune cells (61). Up to now, studies about the correlation between NAC and RA are still rare. A clinical trial conducted recently showed that NAC supplementation could reduce the levels of several mediators involved in oxidative stress, but could not reduce disease activity or improve the symptoms of RA patients (62).

### Natural antioxidants in plants

Quercetin is an ingredient widely existing in various plants. It has been proven to have numerous protective effects such as antioxidation, reducing inflammation and preventing cancer (63). Recently, a clinical study demonstrated the function of quercetin in ameliorating inflammation and improving symptoms in RA patients (64). Experiments based on animal models further confirmed this and uncovered the fundamental mechanisms (63). Firstly, quercetin could reduce chemokines and pro-inflammation cytokines, thus inhibiting neutrophil infiltration. In addition, quercetin could also increase apoptosis and inhibit the release of pro-inflammatory cytokines by macrophages. Moreover, quercetin could inhibit autophagy and reduce the production of NETs (65–68).

Resveratrol is another ingredient extracted from numerous plants. Animal studies suggested that resveratrol was able to reduce ROS and alleviate RA (69). A meta-analysis of preclinical models showed that resveratrol could decrease the level of several pro-inflammatory cytokines including IL-1, IL-6 and TNF-α (70). The beneficial effect of resveratrol in RA patients was also verified by a clinical trial, where those accepted daily resveratrol supplementation showed improved clinical symptoms and serum inflammation indicators (71).

Icariin is the major ingredient of *epimedium*, a traditional herb in China. Evidence accumulates that icariin is able to alleviate inflammation and regulate immunology (72). Experiments based on arthritis rat models showed that it could decrease the levels of pro-inflammatory mediators, reduce the density of neutrophils and suppress joint degradation (73).

Moreover, Tetrandrine, a kind of alkaloid separated from *Stephania tetrandra* S. Moore, was able to mitigate the symptoms of RA in arthritis murine models. Not only could it decrease serum IL-6 level, but it was also capable to inhibit NETs formation (74). Cedrol, which can be found in ginger, was also considered able to attenuate inflammation in RA. It was verified by animal models that cedrol could inhibit the phosphorylation of JAK3 protein, thus inhibiting the secretion of pro-inflammatory cytokines, and decreasing the neutrophil count (75).

## Discussion

**Figure 1:**
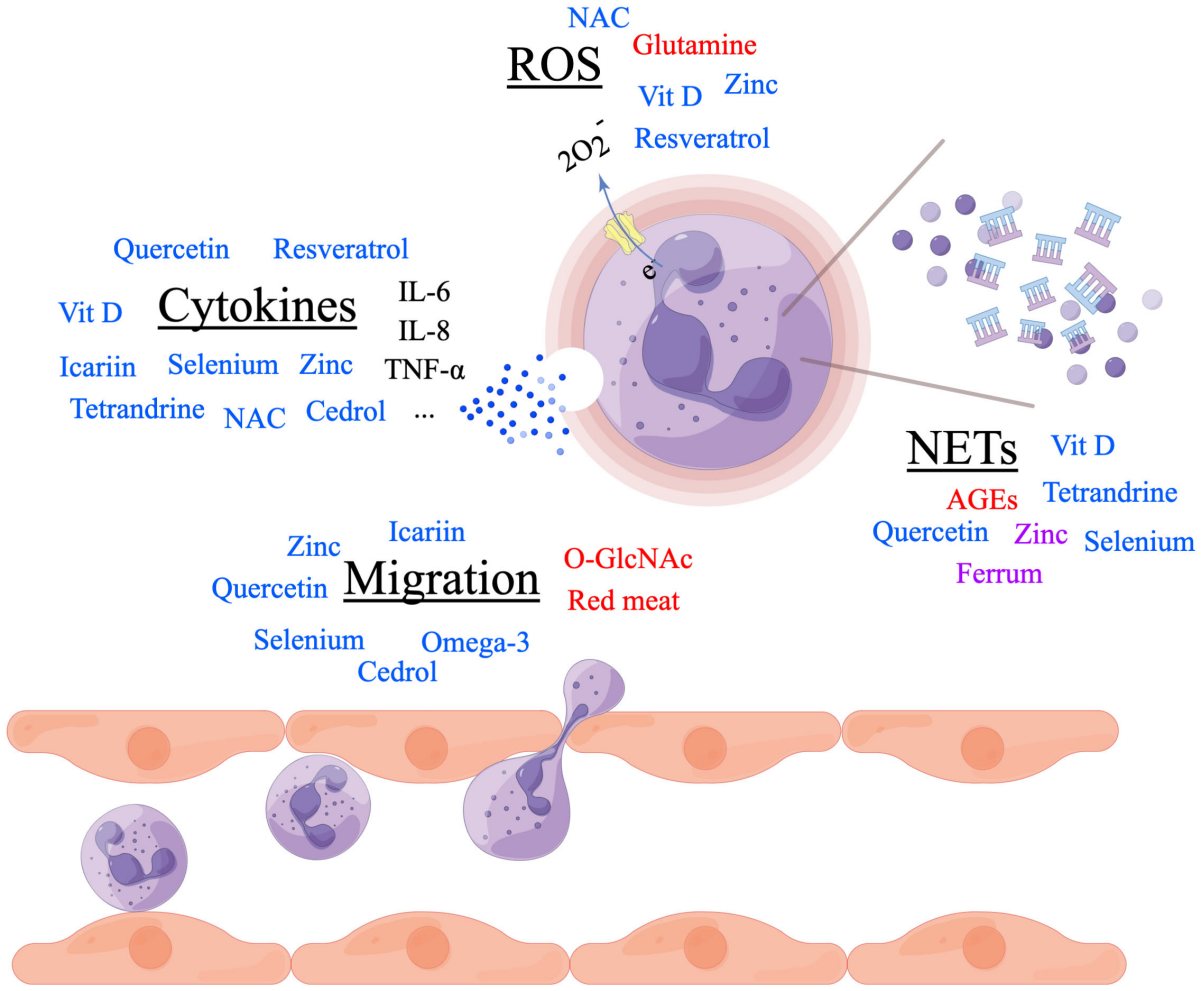
Nutrients in regulating rheumatoid arthritis (RA) through neutrophils. Nutrients in red font promote the process. Nutrients in blue font inhibit the process. Nutrients in purple font controversially influence the process. Made by Figdraw.

The aetiopathogenesis of RA was quite complicated with both genetic risk factors and environmental risk factors involved. In the past decades, more and more researches focused on nutrients in RA and revealed its important role in the prevention and treatment of RA. In this study, we summarized the function of several macronutrients and micronutrients in regulating the onset and disease severity of RA through modulating the migration and activity of neutrophils (**Table 1**). Although controversies existed on the effects of red meat, zinc, Ferrum, NAC and so on, additional sugar intake and excessive energy consumption were widely accepted as risk factors of RA, and omega-3 polyunsaturated fatty acid, vitamin D supplementations, selenium, as well as ingredients extracted from plants, showed their promising effects on prevention of RA onset and amelioration of disease severity (**Figure 1**). Whatever, a healthy diet with more vegetables and fruits as well as less red meat and sugar was recommended in RA.

**Table 1:** Summarization of nutrients in regulating rheumatoid arthritis (RA) through neutrophils.

Nutrients	Influence to neutrophils
Nutrients exacerbate inflammation	
Carbohydrates (especially monosaccharide)	Lead to increased body weight and elevate the risk of RA. Promote the production of advanced glycation end products and enhance autophagy and NETosis.
Glutamine	Participant in producing ROS as the substrate of NADPH. Promote the synthesis of O-GlcNAc and increase neutrophil mobility.
Red meat	Associated with higher neutrophil counts and earlier disease onset.
Nutrients ameliorate inflammation	
Omega-3 fatty acid	Suppress chemotaxis through inhibition of NF-κB signal.
Vitamin D	Reduce pro-inflammatory mediators, inhibit the release of ROS, and decrease NETosis.
Zinc	Decrease the release of pro-inflammatory mediators and ROS by epigenetic mechanisms. Controversial role in the function to NETosis.
Selenium	Reduce neutrophil counts, decrease NETs production and downregulate pro-inflammatory cytokines.
Ferrum	Both iron deficiency and iron overload will promote NETosis.
N-acetylcysteine	Remove ROS and inhibit the synthesis of pro-inflammation cytokines.
Quercetin	Suppress chemotaxis, reduce cytokines and inhibit NETs production.
Resveratrol	Decrease inflammatory cytokines and reduce ROS production.
Icariin	Decrease the levels of pro-inflammatory mediators, reduce the density of neutrophils and suppress joint degradation.
Tetrandrine	Decrease serum IL-6 level and inhibit NETs formation.
Cedrol	Decrease pro-inflammatory cytokines and reduce neutrophil count through inhibiting the phosphorylation of JAK3 protein.

## Author contributions

Y-RS contributed in literature search and manuscript writing. D-YX revised the manuscript. JL raised the idea for the article and proofread the manuscript. All authors contributed to the article and approved the submitted version.
